# Pregnancy characteristics and adverse outcomes in offspring of women with epilepsy: a prospective registry study from Mainland China

**DOI:** 10.3389/fneur.2023.1195003

**Published:** 2023-08-11

**Authors:** Rui Li, Qian Chen, Xing Cao, Hua Yan, Pei Wang, Qun Huang, Xiaoyi Li, Fang Chen, Yangchao Li, Qingxia Kong, Chonglun Guo, Qi Zhang, Qiulei Hong, Yong Liu, Xiaoli Xiong, Yanbing Han, Xiaohua Xiao, Kuiyun Wang, Xunyi Wu, Xi Zhu, Qing Zhang, Lei Chen

**Affiliations:** ^1^Department of Neurology, West China Hospital, Sichuan University, Chengdu, Sichuan, China; ^2^Department of Clinical Research Management, West China Hospital of Sichuan University, Chengdu, Sichuan, China; ^3^Department of Neurology, Affiliated Hospital of North Sichuan Medical College, Nanchong, Sichuan, China; ^4^Department of Neurology, Jianyang People's Hospital, Chengdu, Sichuan, China; ^5^Department of Neurology, Xianyang First People's Hospital, Xianyang, Shanxi, China; ^6^Department of Pediatrics, The WenJiang Maternal and Child Health Hospital, Chengdu, Sichuan, China; ^7^Department of Neurology, Guizhou Provincial People's Hospital, Guiyang, Guizhou, China; ^8^Department of Neurology, Panzhihua Central Hospital, Panzhihua, Sichuan, China; ^9^Department of Neurology, The First Affiliated Hospital of Dali University, Dali, Yunnan, China; ^10^Department of Neurology, Affiliated Hospital of Jining Medical College, Jining, Shandong, China; ^11^Epilepsy Center, Suichuan County People's Hospital, Suichuan, Jiangxi, China; ^12^Department of Neurology, Hospital of Chengdu Office of People's Government of Tibetan Autonomous Region, Chengdu, Sichuan, China; ^13^Department of Neurology, The First Affiliation Hospital of Xi'an Jiaotong University, Xian, Shanxi, China; ^14^Department of Neurology, Guangyuan Mental Health Center, Guangyuan, Sichuan, China; ^15^Department of Neurology, The First Affiliated Hospital of Kunming Medical University, Kunming, Yunnan, China; ^16^Department of Geriatric Medicine, Shenzhen Second People's Hospital, Shenzhen, Guangzhou, China; ^17^Department of Neurology, The Jintang First People's Hospital, Chengdu, Sichuan, China; ^18^Department of Neurology, Huashan Hospital, Fudan University, Shanghai, China; ^19^Department of Neurology, The Third People's Hospital of Chengdu, Chengdu, Sichuan, China; ^20^Department of Neurology, General Hospital of Ningxia Medical University, Yinchuan, Ningxia, China

**Keywords:** seizure, women with epilepsy, anti-seizure medicine, adverse outcome, valproate

## Abstract

**Objective:**

This study aimed to explore the influencing factors of adverse outcomes in the offspring of women with epilepsy (WWE) and to analyze the changes brought about by the epilepsy knowledge popularization campaign in China (EKPCIC).

**Methods:**

This nested case-control study focused on WWE and their offspring from a female epilepsy cohort in mainland China. From January 2009 to August 2022, WWE was prospectively enrolled in 32 study centers. This study aimed to observe the health outcomes of their offspring within 1 year of age. The main outcome measure assessed the health status of the offspring within their first year of age. We aimed to analyze the effects of seizures, anti-seizure medicines (ASMs), and a lack of folic acid supplementation on adverse outcomes in the offspring of WWE and to explore the changes in perinatal management and adverse outcomes of the offspring after dissemination of the EKPCIC in 2015. Additionally, subgroup analyses were conducted to compare seizure control during pregnancy between the valproate and non-valproate groups.

**Results:**

In total, 781 pregnancies in 695 WWE were included, of which 186 (23.69%) had adverse outcomes. The National Hospital Epilepsy Severity Scale score, number of seizures, status epilepticus, ASM type, and valproate and folic acid doses were associated with a high risk of adverse outcomes. After the EKPCIC, the use of ASMs (*P* = 0.013) and folic acid (*P* < 0.001), the seizure-free rate during pregnancy (*P* = 0.013), and the breastfeeding rate (*P* < 0.001) increased, whereas the incidence of complications during pregnancy decreased (*P* = 0.013). However, there was no significant difference in the incidence of adverse outcomes between the analyzed offspring pre-/post-EKPCIC. Additionally, there was no association between the frequency of seizures at different time points during pregnancy and the use of valproate (*F* = 1.514, *P* = 0.221).

**Conclusion:**

Possible factors influencing adverse outcomes in the offspring of WWE include seizures, type and number of ASM usage, and a lack of folic acid supplementation. Although the management of WWE during pregnancy is now more standardized, further efforts are needed to reduce adverse outcomes in offspring.

## Introduction

Epilepsy is a major public health problem that seriously affects the quality of life of patients and their families. In addition to unpredictable seizures, women with epilepsy (WWE) face challenges related to safe delivery and the health of their offspring during pregnancy. Twenty years ago, countries in Europe and the United States began to conduct registration studies on WWE facing fertility problems (1). Previous registration studies in Europe and the United States have generally shown that the offspring of WWE have adverse outcomes, such as severe major congenital malformations (MCMs), developmental delay, low birth weight, and language delay. These adverse outcomes have been reported to be two to three times higher in children of WWE compared to those born to healthy women (2–4). The factors influencing adverse outcomes in the offspring of WWE are multifaceted, only partially known, and tend to exhibit high heterogeneity across regions or cohorts. Most studies have shown that adverse outcomes in the offspring of WWE are associated with the use of anti-seizure medications (ASMs) during pregnancy (5, 6), especially in combination with high-dose drugs (7, 8). In contrast, other studies have shown that adverse outcomes in this population are associated with seizures, a lack of folate use, and complications during pregnancy (9, 10). Conversely, other studies have shown that these factors do not have a significant effect on future generations (3, 11).

The prescription patterns of ASMs for WWE during pregnancy in China differ from those observed globally (12, 13). In this country, the proportion of patients with epilepsy (in both men and women) who discontinue their medications on their own is among the highest in the world (14, 15). Our previous research has also shown that approximately one-third of WWE initiate pregnancy when their epilepsy is not controlled, leading to a higher rate of seizures during pregnancy compared to those reported in international reports (16). Thus, these factors may be associated with poor outcomes in their offspring.

Previous Chinese studies have shown that adverse outcomes in the offspring of WWE are mainly associated with unpredicted pregnancies and complications during pregnancy (13, 17); however, the above studies mainly focused on Beijing, Zhejiang, Taiwan, and other provinces and cities, and most were single-center reports. In 2015, the Department of Neurology of West China Hospital of Sichuan University led a nationwide initiative to promote epilepsy guidelines and conducted widespread education programs targeting patients with epilepsy (18). Therefore, the purpose of this study was to explore the influencing factors of adverse outcomes in the offspring of WWE based on the Women with Epilepsy of Child-bearing Age Ontology (WWECA). We also aimed to analyze the impact of standardized management of WWE.

## Methods

### Study design

A prospective cohort study entitled “Construction and Application of the WWECA” was previously conducted, with West China Hospital of Sichuan University as the main center. This study involved the collaboration of 31 medical institutions from various regions in China and was registered with the Chinese Clinical Trial Registry (ChiCTR2100046318). WWE of childbearing age (20–45 years old) who signed informed consent forms were enrolled and followed up from January 2009 to August 2022 in Sichuan, Yunnan, Shanxi, Guizhou, Jiangxi, Ningxia, Fujian, and other regions. More than 6,000 cases were registered in this cohort.

In this study, a nested case-control study was established based on the WWECA cohort. The case group consisted of WWE offspring with adverse outcomes, while the control group consisted of WWE offspring without adverse outcomes. This study aimed to report the distinct characteristics of these groups and explore possible influencing factors. This study was approved by the Biomedical Ethics Committee of the West China Hospital of Sichuan University and the ethics committees of the participating centers in China (ethical approval number: No. 799, 2019).

### Study criteria

All pregnant participants in the WWECA cohort received regular follow-ups every 3 months by a multidisciplinary team (MDT) of epilepsy specialists and obstetricians. After delivery, the offspring of the WWE were followed up by pediatricians. The inclusion criteria were as follows: (1) female participants aged 20–45 years, (2) diagnosed with epilepsy by two or more epilepsy specialists based on the International League Against Epilepsy Diagnostic Criteria for Epilepsy (2014) and Classification Criteria (2017) (19, 20), and (3) pregnancies between 2009 and 2022 after the diagnosis of epilepsy. The exclusion criteria were as follows: (1) intelligence quotient (IQ) < 70, (2) history of psychogenic non-epileptic seizures, (3) presence of progressive brain disease or other significant illness, (4) a voluntary termination of pregnancy of the mother's personal choice and unrelated to fetal health, and (5) refusal to participate in the study or withdrawal of consent during follow-up. The follow-up endpoint extended from the completion of delivery until the offspring reached 1 year of age or in the event of a miscarriage.

### Information registration and evaluation

The following information on the included patients was registered by doctors and trained researchers: (1) demographic characteristics, including ethnicity, place of residence, education, age at pregnancy, birth history, and family history of epilepsy and malformations. (2) Epilepsy characteristics, including age at onset, course of epilepsy, history of epilepsy surgery, drug-resistant epilepsy, type of seizures, and seizures from 9 months before pregnancy to the end of pregnancy (number, type, duration of epilepsy, and severity). According to the history of epilepsy at the time of enrollment, drug-resistant epilepsy was defined as the failure of adequate trials of two tolerated, appropriately chosen, and used antiepileptic drug schedules (whether as monotherapies or in combination) to achieve sustained seizure freedom (21). Seizure severity was calculated using the National Hospital Epilepsy Severity Scale (NHS3) (22). Given that the duration of pregnancy is generally 9 months, we compared the number of seizures before (N1) and during the 9 months of pregnancy (N2). Worsening of seizures during pregnancy was defined as N2 ≥150% N1, improvement as N2 ≤ 50% N1, and stability otherwise. (3) Use of ASMs and folic acid supplementation during pregnancy. (4) Pregnancy and perinatal outcomes of WWE and the health status of their offspring, including pregnancy complications, delivery methods, feeding methods, and the growth and development of their offspring from birth to 1 year of age.

### Outcome indicators and evaluation

In this study, adverse outcomes in the offspring of WWE within 1 year of age were used as outcome indicators. Offspring health was assessed by an MDT, composed mainly of pediatricians and pediatric neurologists. The following adverse outcomes were considered: (1) spontaneous fetal loss (termination of pregnancy) and (2) MCMs detected at any stage of screening, including via ultrasound during pregnancy, physical examination at birth, and offspring examination within the first year of life. MCMs refer to defects identified through imaging or clinical examination during the development of body parts or organs that seriously affect a child's quality of life or require surgical intervention or close monitoring and follow-up (23); (3) premature birth (i.e., gestational age < 37 weeks); (4) low birth weight (i.e., birth weight < 2,500 g); (5) assessment of neuropsychiatric development delay at 1 year of age using the Child Neuropsychological Development Scale-Revised 2016 (CNBS-R2016), with a score less than two standard deviations from the mea*n* (< 70) (24); (6) compound adverse outcome −1: severe congenital malformation or neuropsychiatric development delay (one or more); and (7) compound adverse outcome −2: total adverse outcome (one or more of the above adverse outcomes).

### Statistical methods

The overall demographics, epilepsy characteristics, pregnancy and perinatal outcomes, and offspring health of the enrolled participants were analyzed. Quantitative variables were represented as means and standard deviations, and categorical variables were described as counts and percentages. Then, a flow chart was drawn according to the occurrence sequence of adverse outcomes to find the development law and the inherent prediction function of adverse outcomes.

Due to the severity of adverse events in children and the psychological impact on the mother, we believe that a conservative approach should be taken when analyzing factors associated with adverse offspring outcomes and that whenever univariate analysis suggests a possible risk, it should be of concern to us. We used univariate analysis to evaluate differences in seizures, ASM use, and folic acid use between the healthy offspring group (control group) and the group with adverse outcomes to explore potential risk factors affecting offspring outcomes. The differences between groups were analyzed using the Student's *t*-test or one-way analysis of variance (ANOVA) for quantitative variables and the chi-square test or Fisher's precision probability test for categorical variables. Correlations were evaluated using Spearman's correlation coefficient for quantitative variables and the Kappa identity test for categorical variables. Odds ratios (ORs) and 95% confidence intervals (CIs) were calculated. To further explore the effects of ASM exposure *in utero*, we compared the incidence of adverse events in offspring born to mothers taking different ASMs and plotted the combination.

We classified WWE into two groups according to the time of pregnancy: before EKPCIC (2009–2015) and after EKPCIC (2016–2022), taking into account that EKPCIC occurred in 2015. This allowed us to determine whether the fertility risks WWE encountered and adverse offspring outcomes had improved after EKPCIC. The incidence of ASM and folic acid use, seizure control, maternal complications, and adverse effects were compared between the two groups.

In the subgroup analyses, we used repeated-measures ANOVA to describe variations in seizure frequency during the following four periods: 9 months before pregnancy, early pregnancy (0–13 weeks), mid pregnancy (14–27 weeks), and late pregnancy (28 weeks delivery) in order to better understand changes in seizure frequency before and during pregnancy. Given the large differences in sample size, WWE in the valproate (VPA) group (*N* = 86) was matched 1:1 with WWE in the non-VPA group (*N* = 695) based on seizure frequency during the 9 months before pregnancy. A repeated-measures ANOVA was used to examine the changes in the frequency of seizures before and during pregnancy, with VPA as a correction factor. If Mauchly's spherical hypothesis test was not satisfied, the Greenhouse-Geisser method was used for correction. SPSS Statistics Version 26.0 was used for statistical analysis.

## Results

### Basic information

In total, 781 pregnancies were included among the 695 WWE who completed follow-ups in 105 cities in mainland China. The overall demographics, epileptic characteristics, pregnancy and perinatal outcomes, and offspring health data of the study participants are shown in Table 1.

**Table 1:** Demographic, epilepsy, maternal and perinatal characteristics, and offspring outcomes of WWE in mainland China.

	**Women with epilepsy**
Total enrolled	695
Total pregnancies	781
**Demographic characteristics**	
Rural areas [*n* (%)]	398 (50.96)
Bachelor's degree or above [*n* (%)]	266 (34.06)
Family history of epilepsy [*n* (%)]	54 (6.91)
Family history of malformations [*n* (%)]	0
**Epilepsy characteristics**	
Onset age, years ± SD	15.4 ± 7.9
Epilepsy duration, years ± SD	11.1 ± 7.5
Drug-resistant epilepsy [*n* (%)]	235 (30.09)
Epilepsy surgery [*n* (%)]	76 (9.73)
**Seizure type [*****n*** **(%)]**	
Generalized	228 (29.19)
Focal	477 (61.08)
Unclassified	76 (9.73)
Seizure with tonic-clonic [*n* (%)]	486 (62.22)
NHS3 during pregnancy, scores ± SD	5.8 ± 6.2
Status epilepticus during pregnancy	32 (4.10)
**Seizure during pregnancy [*****n*** **(%)]**	
No seizure	346 (44.30)
Non-convulsive seizure	150 (19.21)
1–4 times tonic-clonic seizures	99 (12.68)
≥5 times tonic-clonic seizures	186 (23.82)
**ASMs [*****n*** **(%)]**	
None-using	253 (32.39)
Withdrawal	86 (11.01)
ASM monotherapy	278 (35.60)
Levetiracetam	107 (38.49)
Oxcarbazepine	44 (15.83)
Lamotrigine	38 (13.67)
Valproate	31 (11.15)
Carbamazepine	31 (11.15)
Topiramate	7 (2.52)
Others	20 (7.19)
**Polytherapy with two ASMs**	144 (18.44)
Levetiracetam+ oxcarbazepine	29 (20.27)
Levetiracetam+ lamotrigine	27 (18.75)
Levetiracetam+ carbamazepine	8 (5.56)
Including valproate	47 (32.86)
Including topiramate	19 (13.29)
Others	16 ()
Polytherapy with more than two ASMs	20 (2.56)
**Pregnant characteristics**	
Gestational age, years ±SD	26.6 ± 4.3
Gestational age >35 year [*n* (%)]	27 (3.46)
Pregnant complications [*n* (%)]	88 (11.27)
**Folate dose [*****n*** **(%)]**	
< 0.4 mg/day	171 (21.90)
0.4–2 mg/day	527 (67.48)
>2 mg/day	83 (10.63)
Cesarean section [*n* (%)]	492 (63.00)
**Feeding methods [*****n*** **(%)]**	
Breast feeding	286 (36.62)
Artificial feeding	294 (37.64)
Mixed feeding	188 (24.07)
**Adverse outcome [*****n*** **(%)]**	
Compound adverse outcome −1	72 (9.38)
Compound adverse outcome −2	186 (23.84)
Fetal loss	13 (1.66)
Premature birth	70 (8.96)
Low birth weight	76 (9.73)
MCMs	23 (2.94)
Neuropsychiatric development delay	53 (6.79)

WWE, women with epilepsy; NHS3, national hospital seizure severity scale; MCM: major congenital malformation; ASM, anti-seizure medicine; Compound adverse outcome −1, MCM or neuropsychiatric development delay (one or more); Compound adverse outcome −2, Overall adverse outcomes (with any of the above adverse outcomes and above).

**Figure 1:**
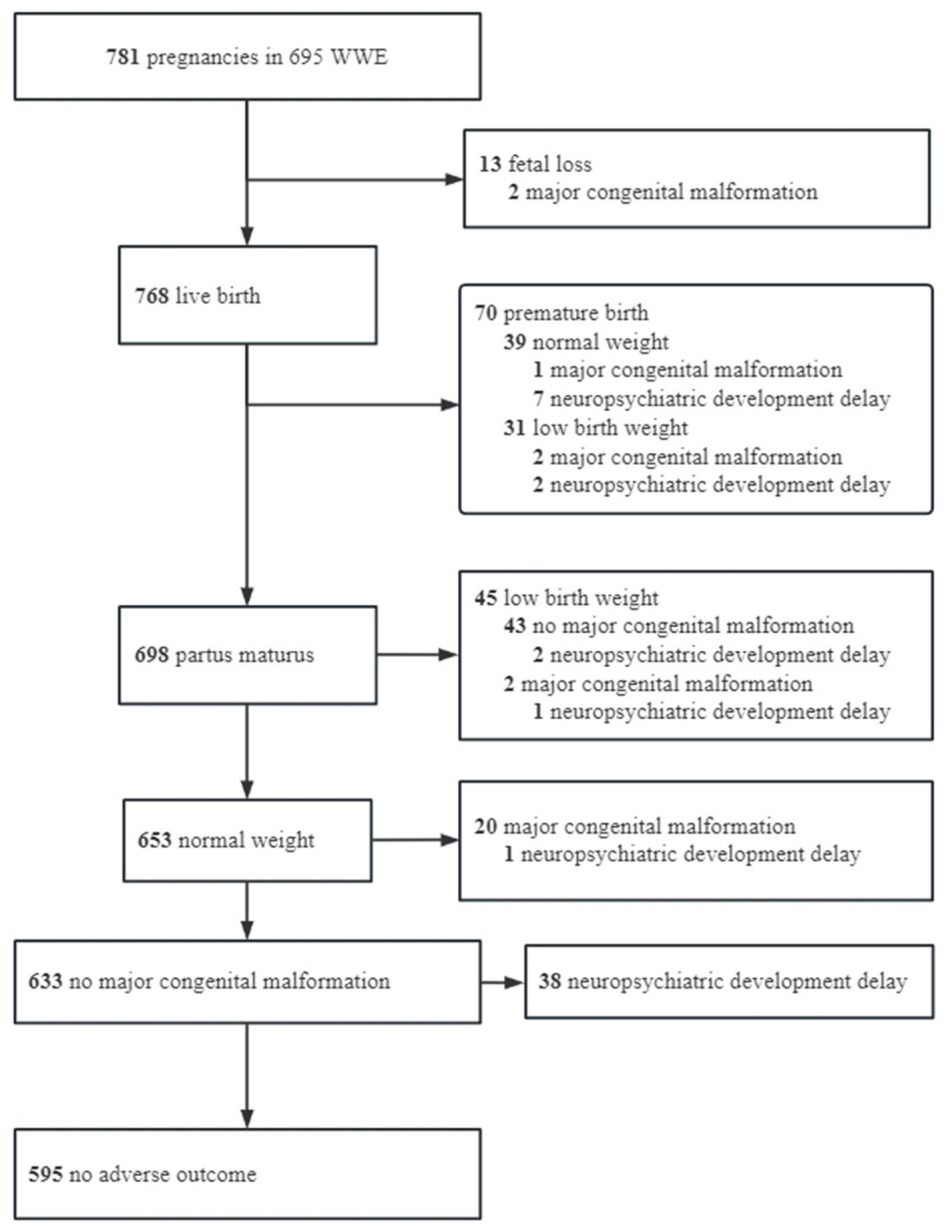
Flowchart of adverse outcome.

Among the 781 pregnancies, 595 (76.18%) had no adverse outcomes within 1 year, 186 (23.82%) had adverse outcomes, and 46 (5.89%) had more than one adverse outcome. Details of the healthy offspring and offspring with adverse outcomes are shown in Figure 1.

The spontaneous fetal loss occurred in 13 (1.66%) pregnancies, including 3 intrauterine stillbirths, 2 cases of MCMs (Appendix 1), 2 cases of fetal loss after status epilepticus, and 6 cases of spontaneous fetal loss of unknown etiology. Nine cases were aborted at 0–12 weeks, and four cases were aborted at 12–28 weeks. Due to the low incidence of fetal loss, no analysis of influencing factors was performed. Excluding fetal loss, there were 70 (9.11%) preterm births, 76 (9.90%) low birth weight, 25 (3.25%) MCMs (Appendix 1), 53 (6.90%) neuropsychiatric developmental delay, and 72 (9.38%) compound adverse outcome −1 (MCM + neuropsychiatric developmental delay). In addition, compound adverse outcome −2 was observed in 186 (23.82%) patients. Further details are listed in Table 1.

### Effects of seizure on adverse outcomes

A total of 385 (49.30%) pregnant women had seizures within 9 months before pregnancy. However, only 6.40% (*n* = 50) of the pregnancies started after seizure-free and ASM withdrawal. A total of 435 (55.70%) pregnant women had seizures during pregnancy, of which 285 (65.51%) were accompanied by generalized tonic-clonic seizures and 186 (42.76%) had more than five generalized tonic-clonic seizures. In addition, status epilepticus occurred in 32 (4.10%) pregnancies. Moreover, there was a higher proportion of drug-resistant epilepsy (*P* < 0.001) and seizures (*P* < 0.001) in these patients within 9 months before pregnancy compared to those who did not develop status epilepticus during pregnancy.

**Figure 2:**
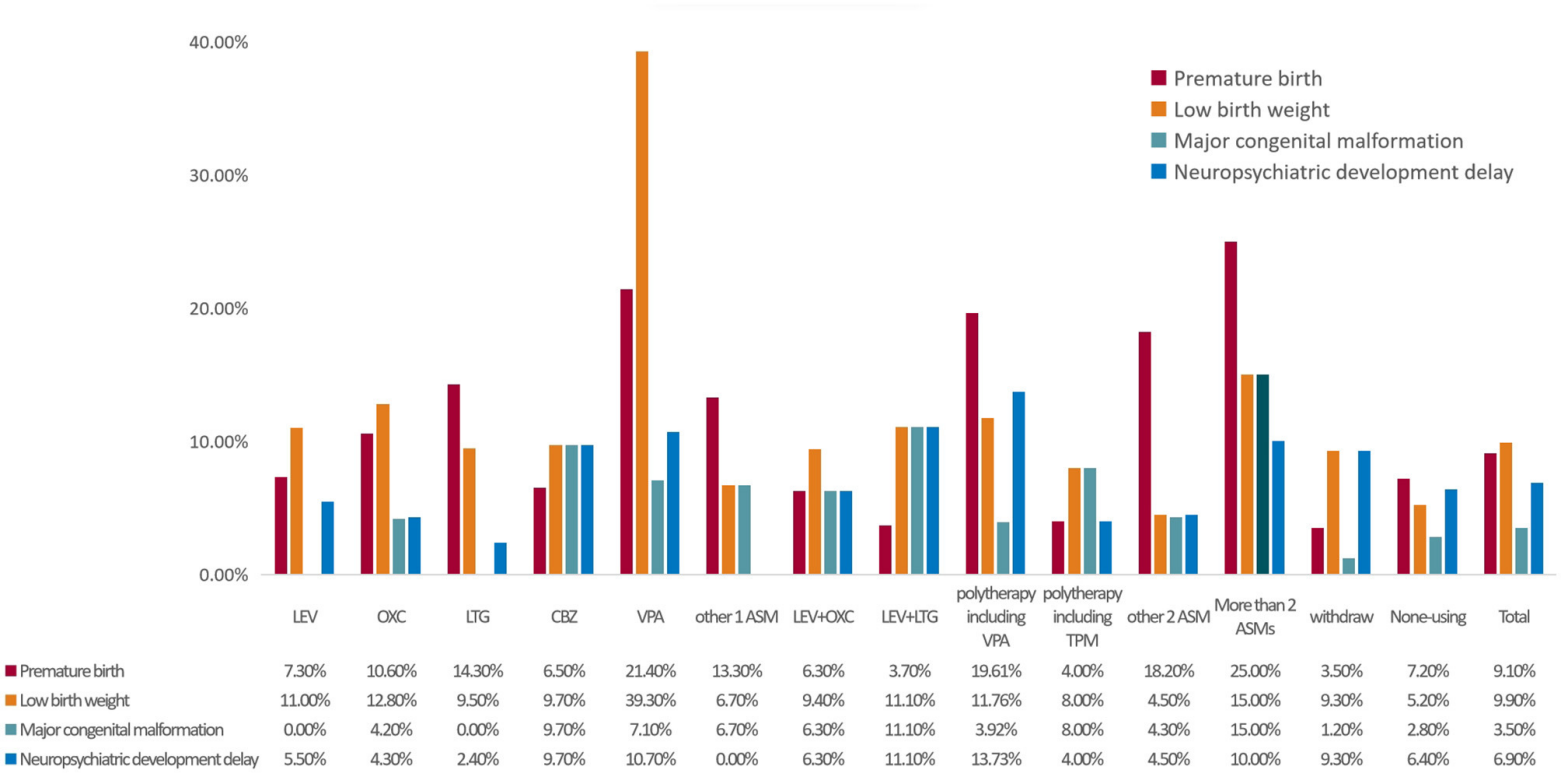
The proportion of adverse outcomes according to ASM. MCM, major congenital malformation; ASMs, anti-seizure medicines; VPA, valproate; LEV, levetiracetam; LTG, lamotrigine; OXC, oxcarbazepine; CBZ, carbamazepine; TPM, topiramate; ASM, anti-seizure medicine. Other 2ASM, LEV+CBZ 8 cases, LEV+OXC 5 cases, others 11 cases.

The patients' seizures worsened in 175 (22.40%) pregnancies, improved in 126 (16.13%) pregnancies, and remained stable in 480 (61.46%) pregnancies. Worsening occurred mainly in the first (34.30%) and third (37.80%) trimesters. Repeated-measures ANOVA showed that the temporal effect of overall seizure frequency was statistically significant (*F* = 4.182, *P* = 0.034; Figure 2A). Notably, the NHS3 score (OR = 1.063) and the occurrence of status epilepticus during pregnancy (OR = 3.542) were found to increase the risk of neuropsychiatric developmental delay. The NHS3 score (OR = 1.064), number of seizures during pregnancy (OR = 1.528), and the occurrence of status epilepticus during pregnancy (OR = 3.662) increased the risk of compound adverse outcome −1. Moreover, the NHS3 score (OR = 1.034) and the occurrence of status epilepticus during pregnancy (OR = 2.982) increased the risk of compound adverse outcome −2 (Table 2). In contrast, age at seizure onset, frequency of seizures during pregnancy, epilepsy surgery before pregnancy, drug-resistant epilepsy, and whether seizures were accompanied by generalized tonic-clonic seizures did not show statistically significant differences in relation to adverse outcomes in offspring.

**Table 2:** Association between seizures during pregnancy and adverse outcomes in offspring.

		**Neuropsychiatric development delay**	**Healthy**	**t/χ^2^**	***P***	**Spearman/Kappa**	***P***	**OR**	**OR 95% CI**	
National hospital epilepsy severity scale		5.32 ± 6.046	7.79 ± 6.957	2.771	0.006	0.100	0.017	1.063	1.017	1.111
Status epilepticus during pregnancy	No	48	578	4.569	0.033	0.090	0.011	3.542	1.252	10.017
	Yes	5	17							
		Compound adverse outcome −1	Healthy							
National hospital epilepsy severity scale		5.32 ± 6.046	7.83 ± 6.819	3.189	0.002	0.118	0.004	1.064	1.023	1.107
Number of seizures during pregnancy	None	23	271	9.063	0.011	0.109	0.005	1.528	1.141	2.046
	1–3 times	18	166							
	More than 3 times	31	158							
Status epilepticus during pregnancy	No	65	578	6.860	0.009	0.097	0.003	3.662	1.464	9.158
	Yes	7	17							
		Compound adverse outcome −2	Healthy							
National hospital epilepsy severity scale		5.32 ± 6.046	6.61 ± 6.559	2.388	0.017	0.081	0.034	1.034	1.006	1.062
Status epilepticus during pregnancy	No	171	578	9.779	0.002	0.073	0.002	2.982	1.459	6.096
	Yes	15	17							

Compound adverse outcome −1, MCM or neuropsychiatric development delay (one or more); Compound adverse outcome −2, Overall adverse outcomes (with any of the above adverse outcomes and above).

### Effects of ASMs and folic acid use on adverse outcomes

A total of 442 (56.59%) pregnancies were treated with ASM during pregnancy, of which monotherapy (*n* = 278, 62.90%) was the most common treatment regimen. A total of 85 (19.23%) pregnancies received VPA or a combination of VPA, and 28 (6.33%) pregnancies received topiramate (TPM) or a combination of TPM (Table 1). Of the pregnancies treated with ASM, 30 were treated with treatment adjustments during pregnancy, of which 18 increased the type or dose of ASM, 10 reduced the type or dose of ASM, and 2 adjusted the type. In total, 86 pregnancies withdrew ASM during pregnancy, 59 (68.60%) withdrew in the first trimester, and the most common reason for withdrawal was fear of the teratogenicity of ASM (58.14%). In addition, 253 pregnancies did not use ASM during pregnancy, and the reasons included never used before pregnancy (49.01%), seizure-free (21.34%), and other (29.64%).

Among pregnant participants who used ASMs, there were higher proportions of individuals with a high income status (*P* < 0.001), individuals with higher educatio*n* (*P* < 0.001), individuals experiencing seizures (*P* < 0.001), and individuals specifically having tonic-clonic seizures (*P* = 0.008) when compared to pregnant participants who did not use ASMs. In addition, treatment with multiple ASMs during pregnancy significantly increased the risk of preterm birth (OR = 1.323) and low birth weight (OR = 1.353). Moreover, VPA use significantly increased the risk of preterm birth (OR = 3.230), low birth weight (OR = 3.141), and neuropsychiatric developmental delay (OR = 2.535) (Table 3).

**Table 3:** Association between ASMs during pregnancy and adverse outcomes in offspring.

		**Premature birth**	**Healthy**	** ${}_{\text{t}/{\text{χ}}^{2}}$ **	***P***	**Spearman/Kappa**	***P***	**OR**	**OR 95% CI**	
Valproate	No	54	545	14.636	< 0.001	0.148	< 0.001	3.230	1.723	6.055
	Yes	16	50							
Number of ASM	None-using	18	205	12.499	0.011	0.096	0.013	1.323	1.072	1.633
	Withdraw	3	67							
	1 ASM	29	207							
	2 ASMs	14	103							
	More than 2 ASMs	6	13							
		**Low birth weight**	**Healthy**							
Valproate	No	59	545	14.623	< 0.001	0.147	< 0.001	3.141	1.703	5.794
	Yes	17	50							
Number of ASM	None-using	13	205	11.909	0.015	0.108	0.005	1.353	1.099	1.666
	Withdraw	8	67							
	1 ASM	37	207							
	2 ASMs	15	103							
	More than 2 ASMs	3	12							
		**Neuropsychiatric development delay**	**Healthy**							
Valproate	No	43	545	5.158	0.023	0.099	0.012	2.535	1.202	5.348
	Yes	10	50							
		**Compound adverse outcome** −**1**	**Healthy**							
Valproate	No	59	545	6.996	0.008	0.102	0.008	2.402	1.233	4.678
	Yes	13	50							
		**Compound adverse outcome** −**2**	**Healthy**							
Valproate	No	150	545	17.344	< 0.001	0.134	< 0.001	2.616	1.643	4.165
	Yes	36	50							

ASM, anti-seizure medicine; Compound adverse outcome −1, MCM or neuropsychiatric development delay (one or more); Compound adverse outcome −2, Overall adverse outcomes (with any of the above adverse outcomes and above).

As shown in Figure 2, the incidence of preterm birth was higher in the >2 ASMs group (25.00%), the VPA group (21.40%), and the polytherapy including the VPA group (19.61%). Additionally, the incidence of low birth weight was high in the VPA (39.30%), >2 ASMs (15.00%), and oxcarbazepine groups (12.80%). Furthermore, the incidence of MCMs was high in the >2 ASMs (15.00%), levetiracetam (LTG) + lamotrigine (11.10%), and carbamazepine groups (9.70%). Finally, the incidence of neuropsychiatric developmental delay was high in the VPA + lamotrigine (13.73%), LTG + lamotrigine (11.10%), and VPA groups (10.70%).

There was a significant correlation between the dosage of folic acid (mg/d) and the overall adverse outcome (*r* = −0.102, *P* = 0.004). This negative correlation indicated that the lower the dosage of folic acid, the more likely the adverse outcome was to occur.

### Changes after the EKPCIC

Compared with WWE before EKPCIC, WWE after EKPCIC had higher rates of ASM use (65.62% vs. 76.39%, *P* = 0.013), folic acid use during pregnancy (73.94% vs. 97.92%, *P* < 0.001), seizure-free pregnancy (34.72% vs. 46.14%, *P* < 0.001), *P* = 0.013), breastfeeding rate (37.50% vs. 67.31%, *P* < 0.001), and lower incidence of pregnancy complications (17.36% vs. 10.06%, *P* = 0.013). However, there was no significant difference in the incidence of adverse outcomes between the two groups (*P* > 0.05).

### Subgroup analyses

**Figure 3:**
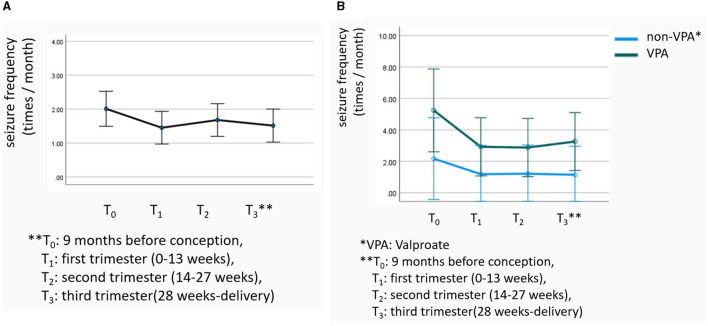
**(A, B)** Differences in seizure frequency changes.

For all WWE, repeated-measures ANOVA showed that the differences in seizure frequency in different periods of pregnancy were statistically significant (*F* = 4.182, *P* = 0.034, Figure 3A). After the VPA group was matched to the non-VPA group, the difference in seizure frequency in different periods of pregnancy between the two groups was still statistically significant (*F* = 9.480, *P* = 0.002), but it was not related to the use of VPA (*F* = 1.514, *P* = 0.221, Figure 3B).

## Discussion

This study represents the largest multicenter, large sample size, and prospective registry of WWE of childbearing age in China. The cohort encompassed different economic and cultural regions in China, taking into account factors that have been found to be associated with adverse offspring outcomes in previous studies, such as demographic characteristics, seizure-related characteristics, ASM use, and folic acid supplementation. This study aimed to provide insights into the maternal and infant health status of WWE in China and to establish a scientific basis for pregnancy management of WWE in Asia. We found that most WWE could conceive successfully and give birth to healthy offspring, with the most adverse offspring outcomes being associated with the number of seizures and seizure status during pregnancy, the specific type of ASM used, and the dosage of folate supplementation. Notably, since 2015, the rates of ASM and folic acid use, seizure-free during pregnancy, and the rate of breastfeeding have increased significantly. Meanwhile, the complication rates during pregnancy have decreased, with no significant change in adverse outcomes in WWE offspring.

### Effects of seizures on offspring

Seizures during pregnancy are harmful to both the mother and fetus because they can cause physical trauma, metabolic stress, fetal heart deceleration, pulmonary edema, aspiration pneumonia, cardiopulmonary arrest, and acute renal failure, among other conditions (25, 26). A Swedish cohort study of 5,373 pregnant women showed that WWE was at a higher risk of pregnancy complications, cesarean sections, and adverse offspring outcomes compared to healthy controls (27). Rauchenzauner et al. (28) showed that generalized tonic-clonic seizures during pregnancy were associated with shortened pregnancy, preterm birth, and an increased risk of low birth weight in boys. Retrospective studies by Adab et al. (9) and Cummings et al. (29) suggested that frequent tonic-clonic seizures during pregnancy were significantly associated with developmental impairment and can be considered independent risk factors for low IQ in offspring. Another study showed that patients with drug-resistant epilepsy who underwent epilepsy surgery before pregnancy had lower ASM exposure, better epilepsy control during pregnancy, and better offspring outcomes (30). These studies suggest that seizures during pregnancy may be associated with adverse outcomes in the offspring. However, no prospective cohort studies in China have demonstrated that seizures during pregnancy are risk factors for adverse health outcomes in offspring.

In our study, we made several important findings. First, we observed a significant association between status epilepticus during pregnancy and neuropsychiatric developmental delay, as well as composite adverse outcomes in offspring. Additionally, we found that two fetuses were miscarried following status epilepticus, while the 10 miscarriages reported by Meador et al. (31) did not show any association with epilepsy findings. This finding highlights the need for further validation in larger sample sizes to confirm this important signal. Simultaneously, we found that more than three generalized tonic-clonic seizures may lead to an increased incidence of adverse outcomes in offspring. Surprisingly, the NHS3 scores appeared to be higher in the healthy group than in the poor outcome group, and it is speculated that seizure duration had the greatest impact on offspring development at the time of seizure. Given that patients with drug-resistant epilepsy and those with seizures 9 months before pregnancy are more likely to develop status epilepticus, more attention should be paid to these patients.

### Effects of ASMs on offspring

The potential adverse effects of ASMs on offspring have been a major concern for pregnant women and a highly discussed topic in research. As early as 1980, VPA was reported to be associated with malformations in offspring, and many subsequent studies have shown that ASM use during pregnancy is associated with miscarriage, MCMs, autism, and neuropsychiatric developmental delays in offspring (32). This risk is particularly prominent with the use of specific ASMs, such as VPA and TPM. Our study supports these observations and suggests that adverse outcomes in the offspring of WWE are primarily associated with the use of VPA and multiple ASMs.

When VPA, either alone or in combination, is used, there is a significantly higher risk of preterm birth, low birth weight, and growth impairment in the offspring. Thus, avoiding the use of VPA during pregnancy appears to be a preferable option. Previous studies have shown that VPA is significantly more effective than other drugs for controlling tonic-clonic seizures in patients with primary generalized epilepsy. However, our study findings reveal an interesting observation: patients who use VPA experience a higher frequency of seizures compared to those who do not, both before and during pregnancy. This suggests that individuals who opt for VPA treatment may have more severe seizures. Therefore, it is crucial to prioritize the development of alternative drugs that can effectively replace VPA in managing seizures.

In this study, the use of multiple ASMs, especially more than two ASMs, was significantly associated with preterm birth and low offspring body weight. Due to the higher economic status and literacy of patients who use ASMs during pregnancy, these individuals generally have better access to resources, and it is assumed that their medications are based on drug-resistant epilepsy. WWE with poor epilepsy control is advised to delay pregnancy while minimizing the number of ASMs used before the pregnancy. Moreover, for patients who face challenges in achieving seizure control, epilepsy surgery may be considered as an option to reduce the need for multiple medications.

This study showed a low incidence of MCMs in offspring treated with LEV or LTG alone; however, a high incidence of MCMs in offspring treated with LEV in combination with LTG was noted, which is inconsistent with previous findings (33). It is important to note that the sample size in our study was small, potentially introducing bias, and therefore, this result should be interpreted with caution. The occurrence of adverse outcomes in offspring is influenced by a combination of many factors, such as the number, type, and dose of ASM. However, due to sample size limitations, we were unable to conduct a more detailed analysis of the effects of specific drug doses. Therefore, further validation with larger sample sizes and longer follow-up periods is necessary to confirm the results of this study.

Another important finding of our study was that approximately one-third of WWE did not take ASMs during pregnancy, which was higher than the 4.22–14.93% reported in other studies (31, 34). The primary reason for not taking ASMs among these women was that they had never used them before. Additionally, WWE who did not use ASMs often had poorer socioeconomic status. These findings suggest that there is still a considerable gap in pregnancy management for Chinese WWE, and special attention should be given to these specific groups. Our study also revealed a significant increase in the use of ASMs during pregnancy after the EKPCIC, which is a manifestation of the success of the epilepsy education program.

### Effects of folic acid use on offspring

Folic acid supplementation during pregnancy reduced the risk of MCMs in offspring, particularly in those with spina bifida. In WWE, evidence for the incidence of congenital malformations with folic acid supplementation during pregnancy is uncertain, but most studies suggest that folic acid supplementation during pregnancy may reduce adverse outcomes, such as preterm birth, spontaneous fetal loss, language delay, and autism (35, 36). While studies have recommended that WWE consume higher doses of folic acid to counteract the effects of ASMs, human and animal experiments have shown mixed results. For instance, recent studies have shown an increased risk of cancer in offspring exposed to high doses of folic acid during pregnancy (2, 37). In our study, we observed that a lower dosage of folic acid taken during pregnancy was associated with a higher likelihood of adverse outcomes in the offspring. However, we did not determine whether the current internationally recommended daily requirement of 0.4 mg of folic acid per day is the optimal dose for female patients with epilepsy to supplement during pregnancy (2). Therefore, the optimal dosage of folic acid supplementation during pregnancy in WWE needs to be further studied, and the effect of folic acid deficiency on offspring neurodevelopment requires long-term follow-up.

### Significance of the EKPCIC

To improve pregnancy management for WWE of childbearing age, we initiated standardized knowledge dissemination and pregnancy education in 2015, which yielded some positive outcomes. Compared to our previous study, we observed significant improvements in seizure-free rates and breastfeeding rates during pregnancy, as well as a reduction in the incidence of complications during pregnancy. However, despite these improvements, the incidence of adverse outcomes in offspring did not decrease. It is speculated that the main contributing factor to this lack of reduction in adverse outcomes is the inadequate management of ASMs during pregnancy. Notably, the number of ASMs used is rising, and 1/5 of WWE treated with ASMs still takes VPA. In addition, with the implementation of measures introduced by the state to reverse the increase in the cesarean section rate, the cesarean section rate in Chinese women dropped significantly to < 40% (38). However, the cesarean section rate in female patients with epilepsy remains higher than 60%. Several factors may contribute to this disparity, including the nature of epilepsy itself and a lack of professional knowledge about epilepsy among healthcare providers (39, 40). Notably, WWE often exhibits greater caution during delivery and tends to opt for cesarean sections as a precautionary measure (40). Furthermore, some studies have indicated an association between seizures during pregnancy and an increased likelihood of undergoing a cesarean section (41).

### Limitations

This study has some limitations. First, considering that previous registry studies have shown a higher incidence of adverse outcomes in WWE than in healthy women, a healthy control population was not established for this study. Second, the follow-up period in this study was relatively short to fully assess adverse neurodevelopmental effects. Third, owing to sample size limitations, we were unable to discuss the effect of ASM dosage on adverse outcomes in the WWE offspring. This aspect will be addressed and refined in future studies with larger sample sizes. Finally, given the unique nature of adverse outcomes, which involve physiological damage to the fetus and psychological impacts on pregnant women, we strongly believe that the protection of this vulnerable population should be improved. As a result, this study prioritized reducing the likelihood of a type II error (false negative) by sacrificing the probability of a type I error (false positive). However, this approach may introduce potential confounding factors that were not included in the analysis, which could impact the interpretation of the results. Therefore, further studies are required to validate and confirm the hypotheses generated from this study.

## Conclusion

This study investigated the clinical characteristics of pregnant WWE and the occurrence of adverse offspring outcomes. We found that the adverse offspring outcomes were mainly related to seizures, the type and number of ASMs, and a lack of folic acid use during pregnancy. After the EKPCIC, the use of ASM and folic acid, the rate of seizure freedom during pregnancy, and breastfeeding rates have increased, and the rate of pregnancy-related complications have decreased. This study provides relevant data on Chinese WWE and their offspring, which further emphasizes the necessity of health education and the importance of strengthening the management of WWE at childbearing age.

## Data availability statement

The original contributions presented in the study are included in the article/Supplementary material, further inquiries can be directed to the corresponding author.

## Ethics statement

The studies involving humans were approved by Biomedical Ethics Committee of the West China Hospital of Sichuan University. The studies were conducted in accordance with the local legislation and institutional requirements. Written informed consent for participation in this study was provided by the participants' legal guardians/next of kin. Written informed consent was obtained from the individual(s), and minor(s)' legal guardian/next of kin, for the publication of any potentially identifiable images or data included in this article.

## Author contributions

RL reviewed the literature, wrote drafts of the manuscript, and prepared the figure. QC performed statistical analyses, adjusted the structure of the article, and participated in the rewriting of some paragraphs during the revision of the manuscript. XC, HY, PW, QHu, XL, FC, YLi, QK, CG, QiZ, QHo, YLiu, XlX, YH, XhX, KW, XW, XZ, and QinZ participated in the article design and data collection. LC supervised the work and provided constructive advice. All authors contributed to the article and approved the submitted version.
