# Supplementary material for: Pregnancy characteristics and adverse outcomes in offspring of women with epilepsy: a prospective registry study from Mainland China
Source: Front Neurol. 2023 Aug 11;14:1195003. doi: 10.3389/fneur.2023.1195003 (PMC10455922; doi:10.3389/fneur.2023.1195003)
Supplement: Supplementary file 1 [file Table_1.DOCX]

Pregnancy characteristics and adverse outcomes in offspring of women with epilepsy: a prospective registry study from mainland China

European journal of neurology

Rui Li1, Qian Chen2, Xing Cao3, Hua Yan4, Pei Wang5, Qun Huang6, Lei Chen1*

⁎Corresponding author: Lei Chen, Department of Neurology, West China Hospital, Highland Health Joint Research Institute, Sichuan University, No. 37 Guo Xue Xiang, Chengdu, Sichuan 610041, China. E-mail: [leilei_25@126.com](mailto:leilei_25@126.com).

Appendix 1 Details of the offspring major congenital malformation

| Patient | age | Folic acid | Folic acid dosage(mg/d) | anti-seizure medicine during pregnancy(g/d) | Seizure during pregnancy | Complications of Pregnancy | Detailed MCM | Other adverse outcome |
| --- | --- | --- | --- | --- | --- | --- | --- | --- |
| 1 | 33 | Yes | 0.4 | LEV 2、TPM 0.1 | None | No | polydactylism | Without |
| 2 | 24 | Yes | 0.4 | LTG 0.1、LEV 1 | Accompanied by tonic-clonic | No | polydactylism | Neuropsychiatric development delay |
| 3 | 38 | Yes | 0.4 | None-using | None | No | Congenital abnormalities of lung | Premature birth、low birth weight、Neuropsychiatric development delay |
| 4 | 21 | Yes | 0.4 | None-using | None | No | cleft lip and palate | Without |
| 5 | 27 | Yes | 0.4 | LEV 2、OXC 1.2 | None | No | hypospadias | low birth weight |
| 6 | 26 | Yes | 0.4 | Withdraw | Accompanied by tonic-clonic | No | Chondrodysplasia of the larynx | Neuropsychiatric development delay |
| 7 | 31 | Yes | 0.4 | LTG 0.15、VPA 1 | Accompanied by tonic-clonic | No | spina bifida | Without |
| 8 | 31 | Yes | 0.4 | CBZ 0.6 | None | Yes | Cardiovascular malformations | Without |
| 9 | 25 | Yes | 0.4 | VPA 1 | Accompanied by tonic-clonic | No | congenital hydrocephalus | low birth weight、Neuropsychiatric development delay |
| 10 | 25 | Yes | 1.6 | LEV 0.5、LTG 0.05、OXC 0.6 | None | Yes | spinal meningocele | Premature birth、low birth weight、Neuropsychiatric development delay |
| 11 | 20 | Yes | 0.4 | OXC 0.6 | Accompanied by tonic-clonic | No | cleft lip and palate | Without |
| 12 | 27 | Yes | 0.4 | OXC 0.6、VPA 0.5、TPM 0.1 | None | No | Syndactyly of the fingers | Without |
| 13 | 23 | Yes | 0.8 | None-using | None | No | The right ear was atresia | Neuropsychiatric development delay |
| 14 | 27 | No | 0 | LEV 1、LTG 0.1 | Accompanied by tonic-clonic | Yes | Cardiovascular malformations | Without |
| 15 | 34 | Yes | 0.4 | TPM 0.1 | Accompanied by tonic-clonic | No | Cardiovascular malformations | Without |
| 16 | 31 | No | 0 | CBZ 0.2 | None | No | hydronephrosis | Without |
| 17 | 22 | Yes | 0.4 | None-using | Accompanied by tonic-clonic | No | Digestive tract abnormalities | Without |
| 18 | 21 | Yes | 0.4 | None-using | Accompanied by tonic-clonic | No | Cardiovascular malformations | Without |
| 19 | 28 | Yes | 0.4 | None-using | None | No | coloboma lobuli | Without |
| 20 | 27 | No | 0 | None-using | Accompanied by tonic-clonic | No | meningomyelocele | Neuropsychiatric development delay |
| 21 | 33 | Yes | 0.4 | LEV 1、OXC 0.6 | Accompanied by tonic-clonic | No | Cardiovascular malformations | Without |
| 22 | 27 | Yes | 0.4 | OXC 0.45 | None | No | Malformation of limb | Fetal loss |
| 23 | 34 | Yes | 0.4 | LTG 0.1、PB 0.045 | None | No | Cardiovascular malformations | Fetal loss |
| 24 | 23 | Yes | 0.4 | VPA 3 | Accompanied by tonic-clonic | No | cleft lip and palate | Premature birth |
| 25 | 26 | Yes | 0.4 | LEV 1-1.75、LTG 0.15 | Accompanied by tonic-clonic | Yes | Cardiovascular malformations | Without |
| 26 | 27 | Yes | 0.4 | CBZ 0.7 | Accompanied by tonic-clonic | No | Skull malformation | Neuropsychiatric development delay |
| 27 | 24 | Yes | 0.4 | LTG 0.2、LEV 1.5、clonazepam 0.001 | Accompanied by tonic-clonic | Yes | Cardiovascular malformations | Without |

Abbreviations: MCM = major congenital malformation; ASMs = anti-seizure medicines; VPA = valproate; LEV = levetiracetam; LTG = lamotrigine; OXC = oxcarbazepine; CBZ = carbamazepine; TPM= topiramate.
